# Physicochemical Properties and Application of Silica-Doped Biochar Composites as Efficient Sorbents of Copper from Tap Water

**DOI:** 10.3390/ma16072794

**Published:** 2023-03-31

**Authors:** Sebastian Drużyński, Krzysztof Mazurek, Urszula Kiełkowska, Adriana Wróbel-Kaszanek, Bartłomiej Igliński

**Affiliations:** Department of Chemical Technology, Faculty of Chemistry, Nicolaus Copernicus University in Toruń, 7th Gagarin Street, PL 87-100 Toruń, Poland; k.mazurek@umk.pl (K.M.); ulak@umk.pl (U.K.); adriana@umk.pl (A.W.-K.); iglinski@chem.umk.pl (B.I.)

**Keywords:** silica-doped biochar, hydrothermal modification, metal ion recovery, pyrolysis, drinking water treatment

## Abstract

This article concerns research on new sorption materials based on silica-doped activated carbon. A two-stage synthesis involved pyrolysis of plant material impregnated in a water glass solution, followed by hydrothermal activation of the pyrolysate in KOH solution. The resulting composite can be used as a sorbent in drinking water filters. The proposed method of synthesis enables the design of materials with a surface area of approximately 150 m^2^·g^−1^, whose chemical composition and structure were confirmed by scanning electron microscopy/energy dispersive spectroscopy (SEM/EDS), X-ray diffraction (XRD), thermogravimetry/differential thermal analysis (TG/DTA) and Fourier-transform infrared spectroscopy (FTIR). The sorption properties of the obtained materials were determined relative to copper ions using the batch experiment method. The optimal operating parameters of the obtained materials relative to copper ions are T = 313.15 K, pH = 5, S:L ratio = 4 g·dm^−3^ and t = 120 min. The research shows that the sorption kinetics of copper ions can be described by a pseudo-second-order model. The plotted copper(II) sorption isotherm clearly indicates the Langmuir model. Under optimal conditions, the maximum sorption of copper ions was 37.74 mg·g^−1^, which is a satisfactory result and confirms the possibility of using the obtained material in drinking water filters.

## 1. Introduction

Since the 1990s, there has been a dynamic increase in the number of articles devoted to new materials with sorption properties. In recent years, adsorbents produced from agricultural and industrial waste have gained popularity and wide interest owing to their wide availability, low cost and favourable physicochemical properties. Currently, biochars are an interesting group of materials which, after various chemical and physical modifications, become promising adsorbents for metals, pharmaceuticals, dyes and other impurities present in water [1,2].

The literature describes many hybrid materials, which are doped nanocomposites with specific properties depending on the purpose of a given sorbent. These materials can have the following properties: magnetic (Fe_2_O_3_ doping [3,4,5]), photocatalytic (TiO_2_ doping [6,7]) and alkaline (MgO [8], CaO [9,10] doping). Their surface can be enriched with nitrogen [11], sulphur and oxygen groups [8] to intensify the coordination of metal ions. Noteworthy are composite microgels characterized by very good sorption properties in relation to metallic (Cd and Cr(VI)) and organic pollutants, e.g., dangerous herbicides present in the aquatic environment [12,13,14]. For the sorption of various types of pollutants from the aquatic environment, activated carbon-based sorbents are very popular. Activated carbons can also be used on a large scale in the preparation of drinking water for the public due to their high efficiency and simple handling. In drinking water treatment plants, activated carbon columns are integrated with other treatment processes such as ozonation, oxidation, ultrafiltration membranes, coagulation–sedimentation–filtration and slow sand filters [15,16]. However, in the literature on the subject, there are few items regarding the introduction of silica into the structure of biochar.

The authors [17] obtained biochar doped with silica nanoparticles with a relatively low porosity of the material, characterized by a specific surface area of up to 30 m^2^·g^−1^. Another example of the use of this type of material is the controlled release of fertilising ingredients into the soil [18]. Carbon materials containing silica are very often used to remove heavy metal ions. For Cr(VI) sorption, biochar coated with nano-silica from the thermal conversion of sawdust was used, obtaining a sorption capacity of 88.2 mg·g^−1^ [19]. Another example of the use of silicon-modified carbon materials is cadmium sorption on biochar obtained from rice husks and wood modified with CaSiO_3_ [20]. The authors found that the modification of biochar caused a significant decrease in the specific surface area and an increase in the number of oxygen-containing functional groups (i.e., Si-O, Si-C). This increased the Cd(II) removal efficiency from 23% to 57%. There are few examples of using silica-doped biochars for metal sorption. Silicon, on the other hand, is the main inorganic component of biochars and plays an important role in the removal of various types of impurities; however, the related mechanisms are still insufficiently described.

During the research, three samples of silica-doped biochars were obtained and subjected to controlled dissolution under hydrothermal conditions in a solution of potassium hydroxide. The sorbent samples were characterized in terms of physicochemical properties, while the sorption properties were tested relative to Cu(II) ions. It is suggested that the obtained materials can be used as sorbents in drinking water filters.

Research conducted by the World Health Organization (WHO) indicates that the content of copper ions ranging from hundredths to several mg·dm^−3^ affects human health. According to WHO recommendations, the content of copper ions in drinking water cannot exceed 2 mg·dm^−3^ [21]. Even though copper is an essential trace element and a component of many important enzymes, its excess is toxic to living organisms [22].

Copper is a very popular material for water installations in houses as it is bacteriostatic, prevents the growth of bacteria and does not change the taste and smell of water. However, in old installations, there is a risk of contamination of drinking water with copper ions due to changes in the pH of the water and the natural ageing of the pipes. Ageing is caused by the growth of a passivating layer of copper oxides in the pipe lumen, which may partially dissolve under suitable conditions and increase the concentration of Cu(II) ions in water, leading to its contamination [23,24].

## 2. Materials and Methods

Reagents used during researchwere copper sulphate pentahydrate, ≥99.0 wt% (Avantor Performance Materials Poland S.A.); sulphuric acid, ≥96 wt% (Avantor Performance Materials Poland S.A.); potassium hydroxide ≥99.0 wt% (Avantor Performance Materials Poland S.A.); and potassium silicate 30 wt% (Avantor Performance Materials Poland S.A.). All were of analytical purity grade. The waste rapeseed cake came from Prem-Vit Sp. J. Inowrocław, Poland.

To characterize the solid phase, the following apparatuses were used: scanning electron microscope Quanta 3D FEG (SEM) FEI Company (Hillsboro, OR, USA); TA Instruments (New Castle, DE, USA) SDT 2960 (TGA-DTA), and the scanning electron microscope model 1430 VP produced by LEO Electron Microscopy Ltd. (Cambridge, UK) For the EDX, Micromeritic’s (Norcross, GA, USA) sorptomat Gemini VII (BET), was used, and FTIR was conducted using Bruker’s (Ettlingen, Germany) FT-IR Vertex 70V. GBC Scientific Equipment Ltd.’s (Melbourne, Australia) Avanta Sigma atomic absorption spectrometer was employed to determine the concentrations of Cu(II).

The adsorption experiments were conducted in a thermostatic bath constant with the Polystat CC1 thermorelay (±0.1 K). The set temperature was controlled with the use of a mercury thermometer with an accuracy of ±0.1 K.

Elmetron’s multifunctional CX-742 device equipped with Ionode’s Ion44C combination electrode was employed for pH measurements.

### 2.1. Synthesis and Characterization of SiO_2_—Biochar Adsorbents

The organic raw material used for biochar synthesis was defatted rapeseed cake. The material was impregnated in a 3 M potassium silicate solution for 24 h and then dried at 353.15 K, also for 24 h. In the next step, the obtained material was ground and subjected to anaerobic pyrolysis at a temperature of 973.15 K with a heating rate of 10 K·min^−1^ for 1 h. The resulting pyrolysate was treated with 0.1 M hydrochloric acid and then with deionized water until the chloride ions were completely washed off. The preparation was dried at 353.15 K and divided into three parts. Each sample was treated with KOH under hydrothermal conditions at a temperature of 473.15 K and a pressure of 1·10^6^ Pa for 2, 4 and 6 h. The obtained sorbents were rinsed with deionized water to pH = 7 and dried at 353.15 K. In this way, three samples of sorbents were obtained and marked R-SiO_2_-2h, R-SiO_2_-4h and R-SiO_2_-6h.

### 2.2. Testing Adsorption Properties

Adsorption tests were carried out using the batch method for standard solutions containing copper(II) sulphate(VI). The material with the highest Cu(II) ion sorption capacity was initially selected. The tests were carried out without determining the optimal sorption parameters for a solution with a concentration of 250 mg·dm^−3^ and sorbent content in the mixture of 5 g·dm^−3^ for 120 min.

R-SiO_2_-2h had the best sorption properties relative to copper(II) ions and other adsorption tests were performed for this material. The concentration of copper(II) ions ranged from 100 mg·dm^−3^ to 400 mg·dm^−3^. The optimal adsorption parameters of copper(II) ions were tested under the following conditions: pH ranging from 2 to 6 (pH was corrected with sulphuric acid(VI) and potassium hydroxide); temperature ranging from 298.15 K to 323.15 K; sorbent content: 1 g·dm^−3^ to 10 g·dm^−3^; and contact time: 10 min to 180 min.

The appropriate mass of the sorbent was weighed in an Erlenmeyer flask, and then 50 cm^3^ of the solution with the planned concentration of copper(II) ions was measured with a pipette. The flask was tightly closed and stirred continuously for the appropriate time in a thermostat at the planned temperature. After regulating the temperature, the solution was separated from the sorbent by filtering the mixture through a G4-fritted funnel under reduced pressure. The resulting solution, after appropriate dilution, was analyzed using flame atomic absorption spectrometry (FAAS). The kinetics of the adsorption of copper(II) ions was also investigated in this way and equilibrium tests were performed to determine the adsorption isotherm.

### 2.3. Analytical Methods

The Copper(II) ion concentrations in the solutions were determined by atomic absorption spectrometry. The sorbents were characterized by determining the content of ash and silica contained in it and by conducting the carbon hydrogen and nitrogen (CHN), SEM/EDS, XRD, TGA, FTIR and Brunauer, Emmett and Teller (BET) analyses.

### 2.4. Theoretical Background

The efficiency of the adsorption process in relation to copper(II) ions was calculated from Equation (1).

(1) $$A=\frac{c_{0}-c_{e}}{c_{0}}\cdot 100\%$$

where *c*_0_ and *c_e_* are the initial and equilibrium concentrations (mg·dm^−3^) of the analyzed ion, respectively.

The equilibrium capacity of the adsorbent *q_e_* (mg·g^−1^) was calculated according to Equation (2).

(2) $$q_{e}=\frac{\left(c_{0}-c_{e}\right)\cdot V}{m}$$

where *V*—volume of solution (dm^−3^), *m*—mass of adsorbent (g).

In the literature, three kinetic models are most often used to mathematically describe the kinetics of adsorption of metal ions from the solution: the pseudo-first-order kinetics model (PFO Equation (3)), the pseudo-second-order model (PFO Equation (4)) and the intramolecular diffusion model (IPD Equation (5)) [25,26].

(3) $$\log\left(q_{e}-q_{t}\right)=\log q_{e}-K_{1}\cdot t$$

where *q_e_* and *q_t_* indicate the amounts of metal ions adsorbed at equilibrium and at time *t* (min), and *K*_1_ (1·min^−1^) is the constant rate.

(4) $$\frac{t}{q_{t}}=\frac{1}{k_{2}\cdot q_{e}^{2}}+\frac{1}{q_{e}}\cdot t$$

where *q_e_* and *q_t_* indicate the amounts of metal cations adsorbed at equilibrium and at time *t* (min), and *k*_2_ (g·mg^−1^·min^−1^) is the rate constant.

The Freundlich (Equation (5)) and Langmuir (Equation (6)) models are most often used to mathematically describe the adsorption isotherm of metal ions from solutions [27].

(5) $$q_{e}=K_{F}\cdot c_{e}^{1/n}$$

where *q_e_* (mg·g^−1^)—sorption at equilibrium state, *c_e_* (mg·dm^−3^)—concentration of cations at equilibrium state, *K_F_* (mg·g^−1^)—maximum adsorption on the sorbent surface and 1/*n*—constant related to the intensity of the adsorption process.

(6) $$\frac{c_{e}}{q_{e}}=\frac{1}{K\cdot q_{m}}+\frac{c_{e}}{q_{m}}$$

where *q_m_* (mg·g^−1^)—the maximum adsorption on the sorbent surface, *K* (dm^3^·mg^−1^)—constant associated with the adsorption energy.

## 3. Results and Discussion

### 3.1. Adsorbent Characterization

**Figure 1:**
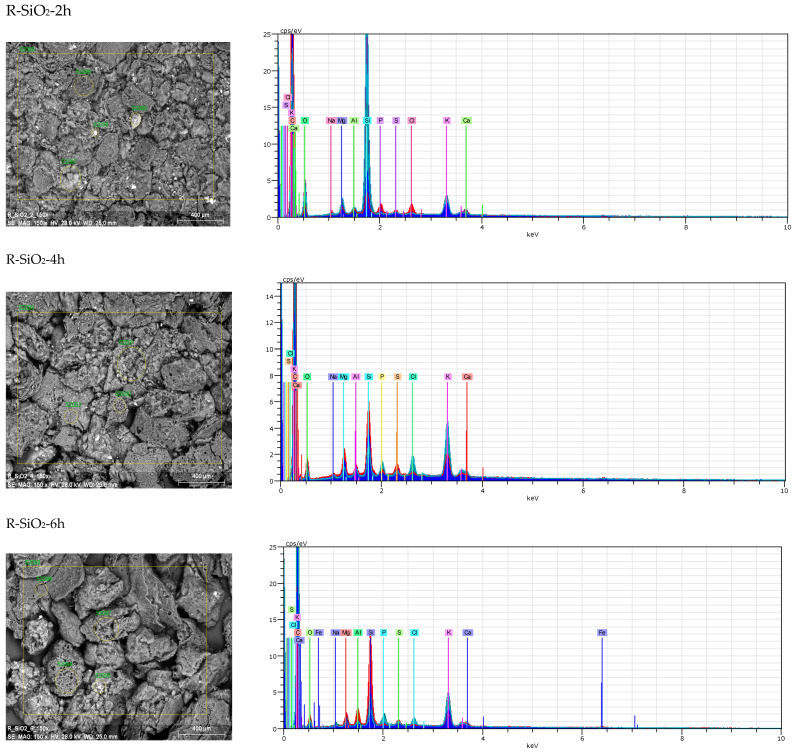
SEM/EDS analysis of the obtained silica-doped biochars.

In the first stage of characterizing the obtained materials, the amounts of carbon, nitrogen, hydrogen, silicon oxide, ash content and bulk density were determined (Table 1), and SEM/EDS analysis was performed (Figure 1). The results indicate that with the increase in the hydrothermal leaching time, the ash content and SiO_2_ content decrease significantly in the tested materials, while the carbon content increases.

**Figure 2:**
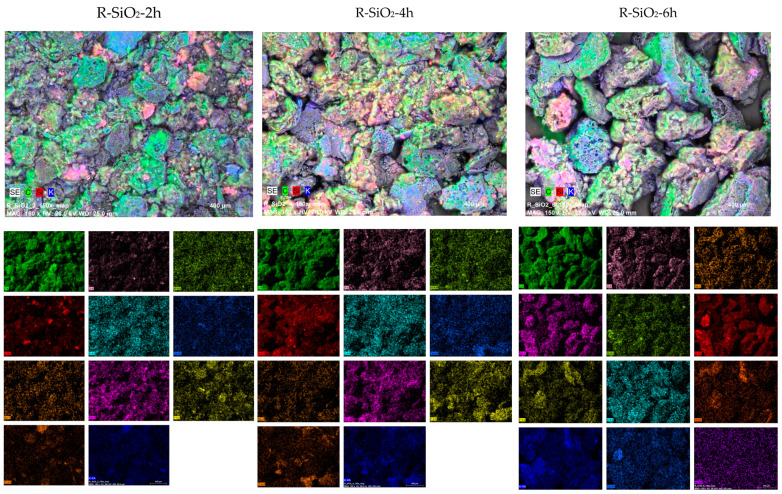
SEM image and mapping results for the biochar–silica composites.

In addition, during the EDS analysis (Figure 1), the following elements that naturally occur in plant material were identified: Na, K, Ca, Mg, Al, P, S and Cl. The maps of the distribution of elements on the surface of the obtained materials (Figure 2) indicate the heterogeneous occurrence of SiO_2_ clusters. The distribution of the remaining elements shows a homogeneous distribution. In the case of the R-SiO_2_-6h sample, the presence of iron was recorded. Considering that this element is absent in the remaining samples, it can be concluded that the sample was contaminated during one of the preparation stages.

The results of the measurement of the specific surface area and porosity of each obtained material are summarized in Table 2. The sorbents are characterized by a relatively low specific surface area, and their pores are wide and have a small volume. The specific surface area of the samples leached in the KOH solution for 4 and 6 h decreased compared with the sorbent modified for 2 h by about 10 m^2^·g^−1^. Extending the modification time from 4 h to 6 h causes no further structural changes on the surface of the silica-doped biochars.

**Figure 3:**
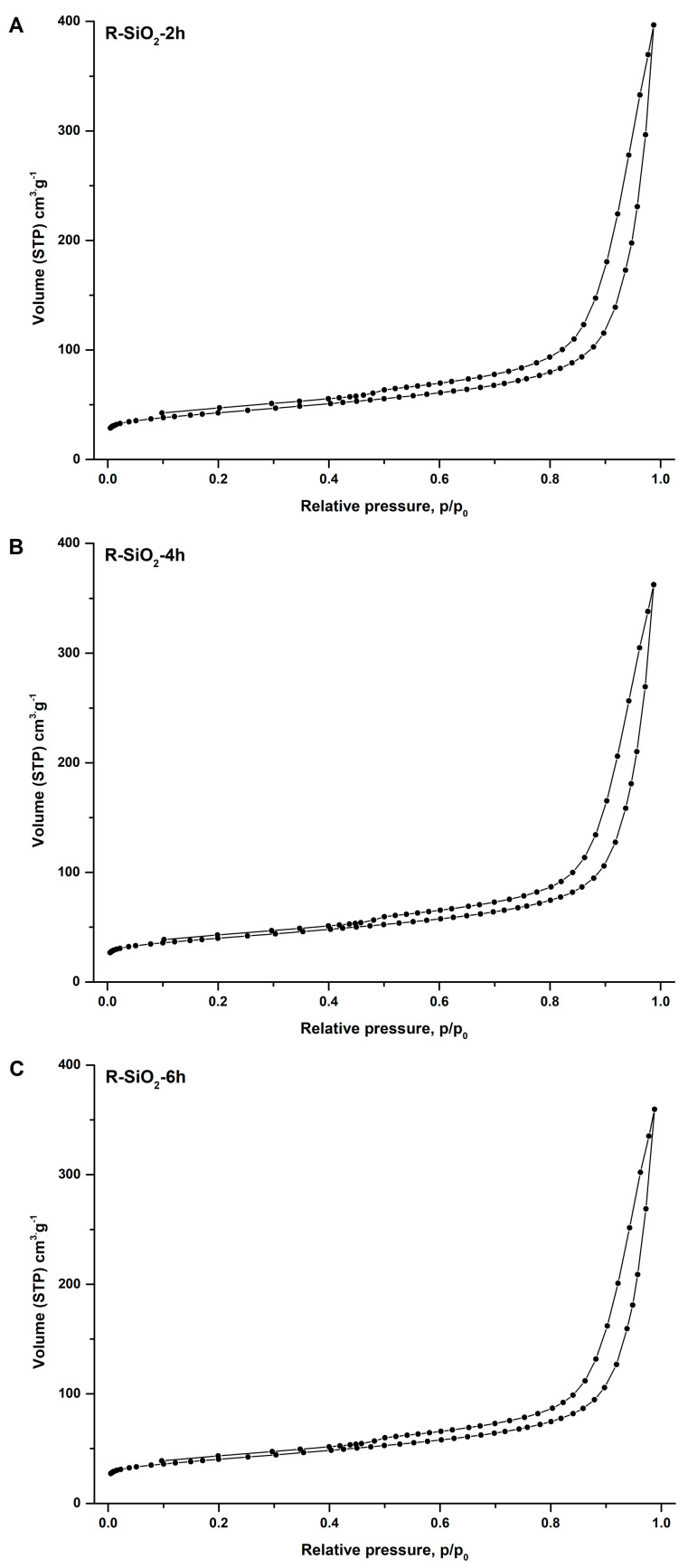
Nitrogen adsorption/desorption isotherms for the biochar–silica composites. (**A**) R-SiO_2_-2h; (**B**) R-SiO_2_-4h; (**C**) R-SiO_2_-6h.

The nitrogen adsorption/desorption isotherms (Figure 3A–C) for all three sorbents look similar and are type II according to the International Union of Pure and Applied Chemistry (IUPAC) classification. This indicates the presence of mesopores calculated according to the Barrett–Joyner–Halenda (BJH) algorithm (Table 2). In Figure 3A–C, a small hysteresis loop is visible in all cases, indicating that the pore size and volume result not only from the free spaces between the packed primary nano- and microparticles but also from the formation of a porous structure inside the sorbent particles, which is consistent with other research results [8,28].

**Figure 4:**
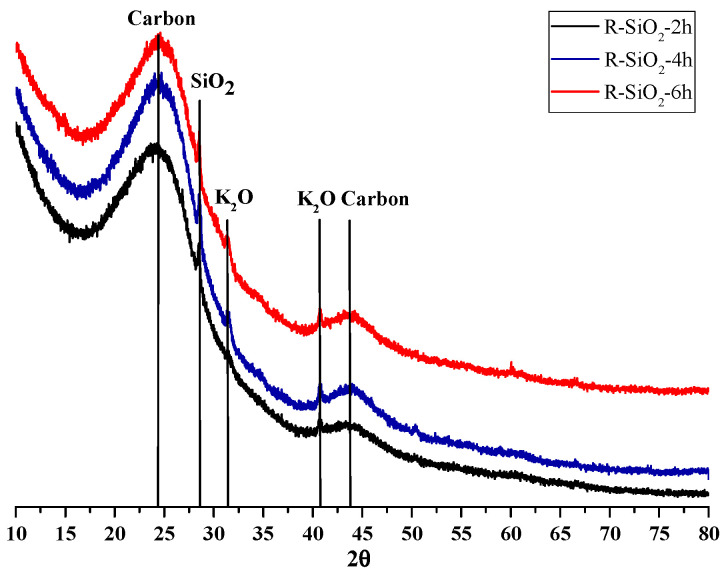
XRD patterns for the biochar–silicates.

Low-intensity reflections are present in the recorded X-ray diffraction patterns (Figure 4), indicating that the obtained sorbent samples are characterized by low crystallinity. Reflections appear at characteristic reflection angles for carbon pyrolysis products (24.44; 43.90°) [29,30], SiO_2_ (28.57°) [30] and K_2_O (31.48; 40.90°) [31].

**Figure 5:**
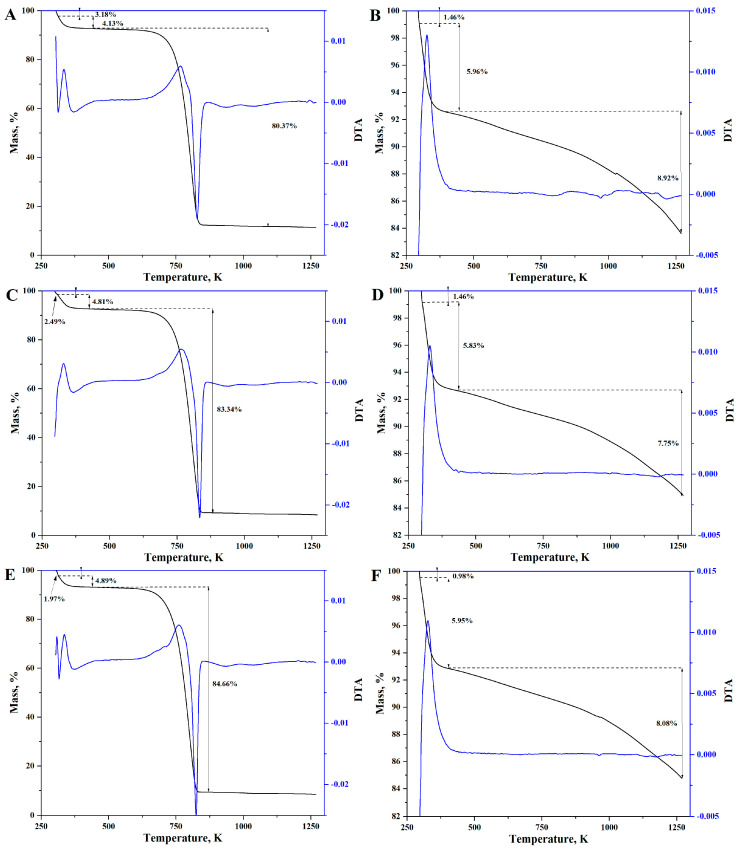
TGA-DTA analysis of biochar–silicon composites; (**A**,**B**) R-SiO_2_-2h in air and nitrogen atmosphere respectively; (**C**,**D**) R-SiO_2_-4h in air and nitrogen atmosphere respectively; (**E**,**F**) R-SiO_2_-6h in air and nitrogen atmosphere respectively.

Figure 5 shows the thermogrammes of the three sorbents obtained. The tests were carried out in the atmosphere of air and nitrogen with a flow of 100 cm^3^·min^−1^ and a heating rate of 5 K·min^−1^. Thermal decomposition of all the samples proceeds in three stages. In the first stage, which lasts until a temperature of 313 K is reached, the samples lose adhesive moisture. The second stage of decomposition (313 K–450 K) is associated with the loss of water adsorbed in the structure of the materials. In the third stage of decomposition (450 K–880 K), in the case of analysis carried out in the air atmosphere, the carbon skeleton of the sample is burned. The TG/DTA analysis performed in the nitrogen atmosphere (Figure 5B,D,F) makes it possible to assess the thermal stability of the obtained materials. The thermogrammes up to a temperature of about 400 K show changes analogous to those obtained during the analysis performed in the air, which is related to the loss of water by the sample. Above this temperature, a slight weight loss of 7% to 9% occurs for all samples, up to a temperature of 1250 K. This phenomenon is associated with further pyrolysis of the samples. The tested materials were obtained at 973.15 K, while the analysis was carried out up to 1250 K.

**Figure 6:**
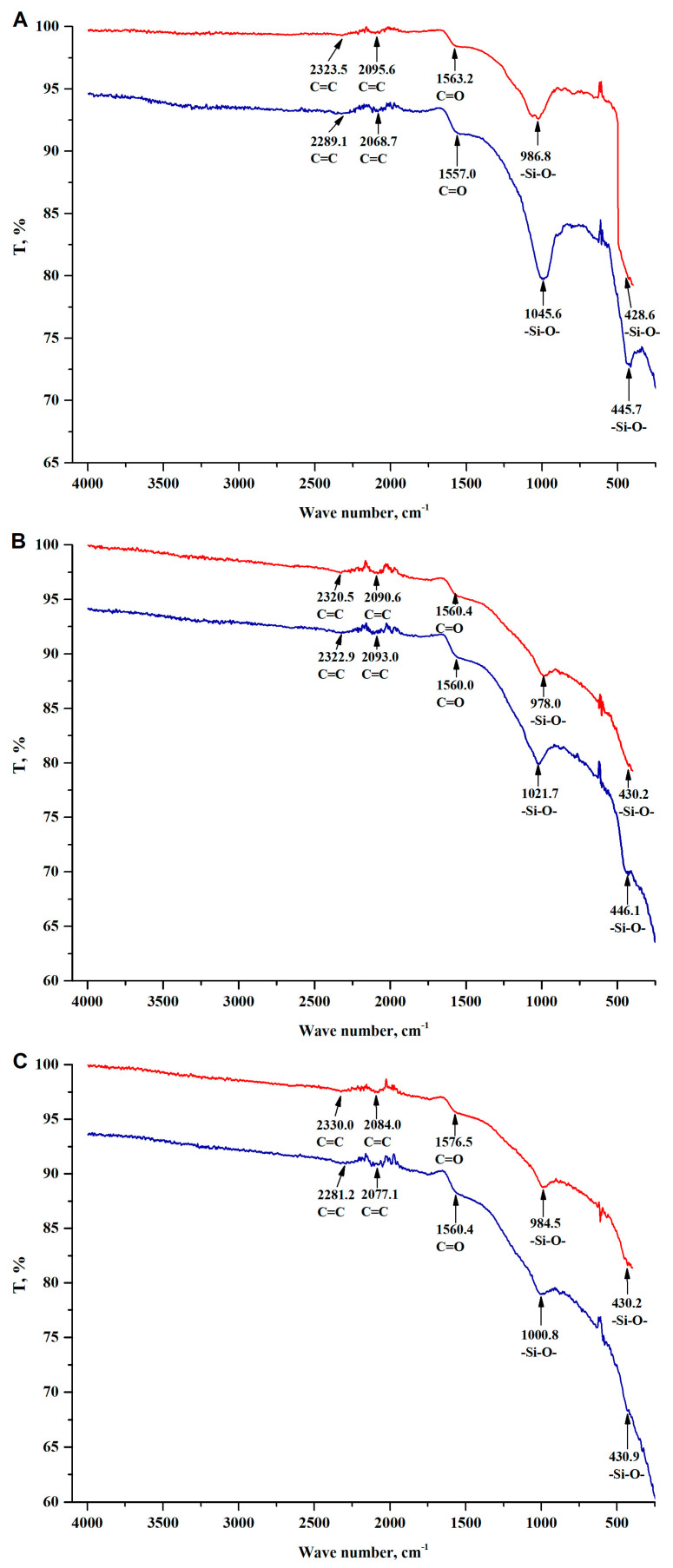
FTIR spectrum of biochars doped with SiO_2_. (**A**) R-SiO_2_-2h; (**B**) R-SiO_2_-4h; (**C**) R-SiO_2_-6h; before sorption—red line; after copper(II) sorption—blue line.

The presence of nitrogen, oxygen or sulphur functional groups on the surface of sorbents significantly improves their ability to coordinate metal ions. Figure 6 shows the FTIR spectra of the obtained sorption materials. The spectra showed low-intensity strands originating from skeletal bonds between carbon atoms (~2300 cm^−1^ and ~2100 cm^−1^) [32,33] and bonds characteristic of silica (~980 cm^−1^ and ~430 cm^−1^) [32,33]. In the spectra (Figure 6A–C), there are no strands characteristic of bonds between silicon and carbon atoms. It should therefore be concluded that silica did not chemically bind to the carbon skeleton during pyrolysis and occurs on the surface in the form of islands (incrustations), which is confirmed by the SEM/EDS test results (Figure 2). Another interesting observation is the lack of bonds characteristic of various types of functional groups (nitrogen, sulphur and oxygen), and in particular for the hydroxyl group. In Figure 6, only a very low-intensity bond of the C=O group was observed at ~1560 cm^−1^ [11]. The lack of a bond characteristic of the hydroxyl group can be explained by the method of preparing the sorbents. As mentioned earlier, after the pyrolysis process, the materials were subjected to hydrothermal modification in a concentrated KOH solution. It should be assumed that the protons in the hydroxyl groups were completely replaced by potassium ions. This observation is confirmed by the EDS results, where a high content of potassium ions was observed in the tested materials.

To assess the effectiveness of the obtained materials in the sorption of metal ions from water or solutions, preliminary tests on the removal of copper(II) ions were carried out under the following conditions: initial concentration of 250 ppm, sorbent content of 5 g·dm^−3^, temperature of 293.15 K and contact time of 2 h. The obtained recovery values for R-SiO_2_-2h, 4h and 6h are, respectively, 41.4, 11.2 and 9.2%. The results indicate that high SiO_2_ content has a decisive effect on the efficiency of copper(II) ion removal from the solution. The biochar subjected to hydrothermal modification for the shortest time of 2 h is characterized by the highest efficiency. This material has the highest silica content. After the sorption process, samples of the solid phase were recovered and FTIR spectra were recorded for them (Figure 6). It was observed that after copper sorption, the bonds characteristic of oxy-silicate groups were more intense and shifted towards higher frequencies. The change in band frequency ranging from 978 cm^−1^ to 1000 cm^−1^ correlates with the hydrothermal leaching time and silica content in the samples. The longer the response time, the lower the content of SiO_2_ and the smaller shifts of the band towards higher frequencies. For the resulting materials leached for 2, 4 and 6 h, the shifts are 58.2; 43.7 and 16.3 cm^−1^, respectively, which is consistent with the research of other authors [34,35]. Given these dependencies, it can be concluded that the majority of copper ions are sorbed by the silica contained on the surfaces of the materials used. Therefore, only R-SiO_2_-2h was used in further sorption studies.

### 3.2. Sorption Properties of R-SiO_2_-2h towards Copper(II) Ions

**Figure 7:**
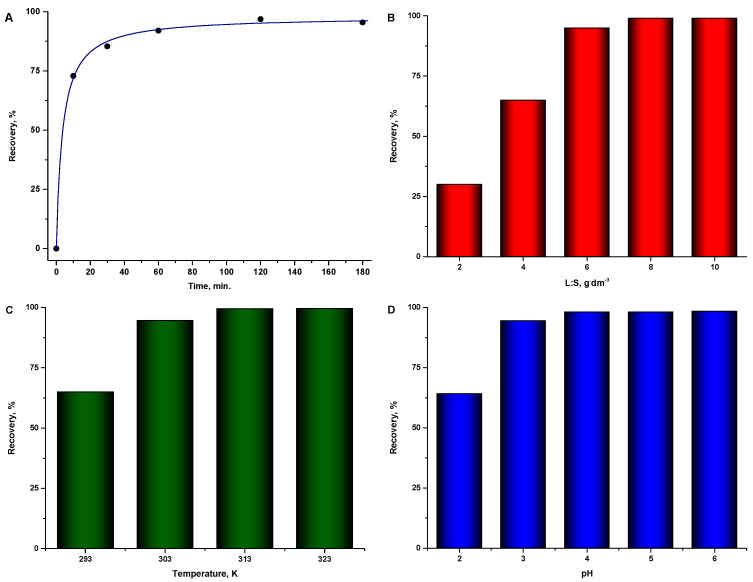
Determination of optimal sorption parameters for copper(II) ions on R-SiO_2_-2h. (**A**) equilibrium time; (**B**) influence of sorbent content; (**C**) influence of temperature; (**D**) influence of pH.

The sorption capacity of metal cations from solutions was determined relative to copper(II) ions. In the first stage of the research, the following optimal sorption parameters were determined: contact time (10 min–180 min), sorbent content in the mixture (L:S—2 mg·dm^−3^–10 mg·dm^−3^), temperature (293.15 K–323.15 K) and initial pH of the solution (2–6). Figure 7A shows the rate at which the state of equilibrium was established between the solution and the solid phase. The tests were carried out at a temperature of 293.15 K, with an initial concentration of copper(II) ions of 50 ppm and a sorbent content in the mixture of 6 g·dm^−3^. The initial pH of the solution was 5.5. It can be seen that the equilibrium is established after 120 min of contact. Another parameter that determines the amount of sorption is the mass of the sorbent relative to the volume of the solution. Figure 7B shows changes in the recovery of copper(II) ions with increasing concentration of the R-SiO_2_-2h suspension. The tests were carried out under the following conditions: T = 293.15 K, time = 120 min, initial concentration of copper(II): 100 ppm, and solution pH: 5.5. Virtually complete recovery of copper(II) ions can be obtained above a sorbent content of 6 g·dm^−3^. Figure 7C shows the tests on the influence of temperature on the amount of copper(II) recovery, which were carried out under the following conditions: initial concentration of copper(II): 140 ppm, L:S = 4 g·dm^−3^, time = 120 min and pH = 5.5. Figure 7C shows an increase in recovery with rising temperature, despite the lower sorbent content in the mixture and the higher initial concentration of copper(II) ions compared with the tests on the contact time and the L:S ratio. The last parameter to be optimized was the initial pH of the solution. The tests were carried out for copper(II) under the following conditions: 180 ppm, L:S = 4 g·dm^−3^, time = 120 min and T = 313 K (Figure 7D). It is noteworthy that the obtained material shows significant sorption in acidic solutions, which was not observed in other studies [8,23]. For solutions with pH > 4, the recovery of copper(II) from the solution is almost complete. In solutions with pH > 6, the sorption of copper(II) on the tested material is impossible owing to its hydrolysis. Further kinetic and equilibrium tests were carried out under the optimal sorption conditions of copper(II) ions on R-SiO_2_-2h, which were: temperature = 313 K, pH = 5–5.5, L:S = 4 g·dm^−3^ and contact time: 2 h.

**Figure 8:**
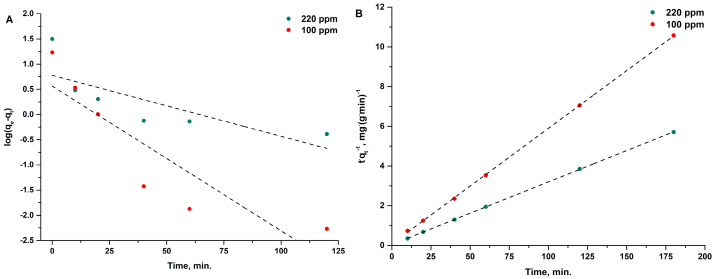
Pseudo-first- (**A**) and second-order (**B**) kinetic fit for adsorption of copper(II) ions onto R-SiO_2_-2h hybrid material. (T = 313.15 K, S:L = 4 g·dm^−3^, pH = 5).

The obtained results of calculations and linear regression parameters are summarized in Table 3. Low values of determination coefficients and very large differences between the experimentally determined ion exchange capacities and those determined from the equation of pseudo-first-order kinetics indicate a low fit of the model to the experimental data, as shown in Figure 8A. The calculated values of the *t*:*q_t_* ratio as a function of sorption time for copper(II) ions for the pseudo-second-order model are shown in Figure 8B. The results of the calculations of the rate constants (*k*_2_) and equilibrium sorption capacity (*q_e_*), as well as the coefficients of determination (R^2^), are presented in Table 3. The data indicate that the pseudo-second-order kinetic model very well describes the kinetics of the adsorption process of copper(II) ions on the sorbent used. This is confirmed by the obtained coefficients of linear determination, which are very high (0.9999). A strong correlation between the calculated and experimental sorption capacities should also be noted.

**Figure 9:**
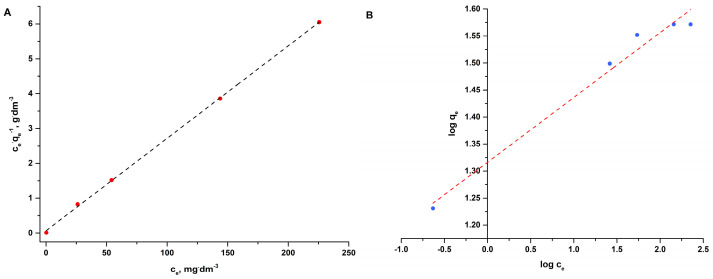
Linear form of Langmuir (**A**) and Freundlich (**B**) isotherms for adsorption of copper(II) ions onto R-SiO_2_-2h hybrid material.

Equilibrium sorption studies of copper(II) ions on R-SiO_2_-2h were carried out for the initial concentration ranging from 100 mg·dm^−3^ to 400 mg·dm^−3^ at a temperature of 313.15 K and a sorbent content of 4 g·dm^−3^ for a contact time of 120 min at a pH of the solution of 5. Freundlich (Equation (5)) and Langmuir (Equation (6)) isotherm equations were used for the mathematical description of the obtained results. The results of the calculations are presented in Table 4 and Figure 9A,B. The coefficients of determination of the rectilinear regression indicate that the Langmuir isotherm model better describes the obtained data on the equilibrium sorption of copper(II) ions.

Comparison of copper(II) sorption onto R-SiO_2_-2h, unmodified biochar from rapeseed cake and silica obtained by hydrothermal synthesis [36] shows the beneficial effect of introducing silica into the sorbent structure. The MCM-41 silica obtained by the authors [36], despite its large specific surface area (839 m^2^·g^−1^) and developed mesoporous structure, shows very poor sorption properties towards copper(II) ions. The determined parameters of the Langmuir isotherm (Table 4) indicate that the proposed method of obtaining and modifying the surface of the composite R-SiO_2_-2h sorption capacity increases 5.5 times (from 6.89 to 37.74 mg·g^−1^) compared with unmodified biochar and 94 times (from 0.40 to 37.74 mg·g^−1^) compared with pure mesoporous silica.

Table 5 contains a comparison of selected maximum adsorption capacities of different types of adsorbents. The calculated maximum sorption capacity of the tested silica-doped carbon sorbent is 37.74 mg·g^−1^. The obtained sorption capacity should be assessed as satisfactory, taking account of the relatively low concentration of copper(II) ions in drinking water, even if the standard is exceeded.

## 4. Conclusions

This article describes the results of research on a hybrid carbon material doped with SiO_2_, which was checked for the possibility of being used as a sorbent for drinking water purification. The sorption material was obtained by pyrolysis of rapeseed cake chemically modified with water glass and hydrothermal activation in KOH solution, which was carried out for 2, 4 and 6 h. All the obtained materials have a specific surface area of 150 m^2^·g^−1^ to 140 m^2^·g^−1^ and an average pore volume Vp ranging from 0.457 to 0.502 cm^3^·g^−1^. The material activated hydrothermally for 2 h had the best sorption properties and also contained the largest amount of silica, which determines the sorption capacity of the material, as confirmed by the FTIR spectra. During the research, the most favourable sorption parameters of copper(II) ions on the sorbent hydrothermally activated for 2 h were determined. The highest values of sorption capacity were obtained for: T = 313.15 K, pH = 5, S:L ratio = 4 g·dm^−3^ and t = 120 min. The equilibrium state for copper(II) ions was reached after 120 min of contact of the sorbent with the solution. The results of the kinetic tests are described by the pseudo-first- and second-order equations. It should be stated that the copper(II) sorption process proceeds in accordance with the pseudo-second-order model.

Langmuir and Freundlich isotherm equation parameters were calculated for the obtained experimental data. The sorption capacity calculation results are closest to reality in the Langmuir model. The determined maximum sorption capacity is 37.74 mg·g^−1^. This value is sufficient to allow use of the obtained material as a sorbent in drinking water filters.

## Figures and Tables

**Table 1:** Chemical composition of silica-doped biochars.

Sorbent	Ash, %	SiO_2_, %	C, %	H, %	N, %	Bulk Density, g·dm^−3^
R-SiO_2_-2h	11.70	2.87	72.58	2.56	4.47	223.0
R-SiO_2_-4h	8.68	2.29	76.70	2.37	4.47	209.7
R-SiO_2_-6h	8.47	1.62	76.87	2.18	4.44	198.1

**Table 2:** Surface area and pore characteristics of biochar–silica composites.

Sample	Surface Area A_BET_ m^2^·g^−1^	Pore Volume cm^3^·g^−1^	Pore Width nm
R-SiO_2_-2h	150.05	0.502	13.38
R-SiO_2_-4h	140.38	0.462	13.35
R-SiO_2_-6h	141.59	0.457	13.41

**Table 3:** Pseudo-first- and second-order kinetic parameters obtained by linear method for adsorption copper(II) on R-SiO_2_-2h hybrid material.

Parameter		Copper(II) Ions Concentration (mg·dm^−3^)			
		First-Order		Second-Order	
Symbol	Unit	100	220	100	220
*q_e_*, exp.	mg·g^−1^	17.02	31.54	17.02	31.54
*k*_1_/*k*_2_	1·min^−1^ or g·mg^−1^·min^−1^	0.0287	0.0121	0.0414	0.0220
R^2^	-	0.7918	0.6130	0.9999	0.9999
*q_e_*, calc.	mg·g^−1^	3.66	5.99	17.20	31.69

**Table 4:** Freundlich and Langmuir isotherms parameters for adsorption of copper(II) ions onto R-SiO_2_-2h hybrid material and unmodified biochar from rapeseed cake.

Sample	Freundlich			Langmuir		
	R^2^	*K_F_* (mg·g^−1^)	n	R^2^	*q_m_* (mg·g^−1^)	K (dm^3^·mg^−1^)
R-SiO_2_-2h	0.9791	20.71	8.33	0.9996	37.74	0.3816
Unmodified biochar	0.8735	12.33	0.233	0.9766	6.89	1.4761
MCM-41 SiO_2_ [36]	0.9735	0.1088	2.17	0.9674	0.40	0.2955

**Table 5:** Adsorption capacities of different adsorbents towards removal of copper(II) ions from aqua solutions.

Adsorbent	*q_m_*, mg·g^−1^	References
HCl-treated clay	83.3	[37]
Green vegetable biochar	75.0	[35]
R-SiO_2_-2h	37.74	This work
Mesoporous silica KIT-6	36.43	[38]
Carrot pulp	32.74	[39]
Biochar	15.7	[40]
Mesoporous silica MCM-41	9.7	[41]
Hardwood biochar	4.39	[42]

## Data Availability

Data sharing is not applicable to this article.
